# Halotolerant *Kocuria* and *Bacillus* species enhanced saline-alkaline stress tolerance in foxtail millet

**DOI:** 10.3389/fpls.2026.1910313

**Published:** 2026-08-19

**Authors:** Chih-Ning Ko, Tan-Yu Lee, Wei-Yi Lin

**Affiliations:** Department of Agronomy, National Taiwan University, Taipei, Taiwan

**Keywords:** *Bacillus*, foxtail millet, *Kocuria*, plant growth-promoting bacteria, saline-alkaline stress

## Abstract

**Introduction:**

Saline-alkaline stress is an increasing threat to agricultural productivity due to climate change and anthropogenic activities. Plant growth-promoting bacteria (PGPB) have emerged as a sustainable approach to enhance crop growth and stress tolerance under adverse environmental conditions. In this study, two halotolerant strains, *Kocuria palustris* Pp13 (Pp13) and *Bacillus aryabhattai* Pp16 (Pp16), isolated from the endosphere of Napier grass, were selected to analyze their stress tolerance mechanisms and evaluate their plant growth-promoting traits under saline-alkaline conditions.

**Methods:**

The stress tolerance and plant growth-promoting traits of Pp13 and Pp16 was evaluated under different salinity and pH conditions. The effects of bacterial inoculation on the growth and stress tolerance of foxtail millet (*Setaria italica* L.) were assessed by analyzing plant growth, osmotic and oxidative stress responses, and ion accumulation under saline-alkaline conditions.

**Results:**

Both Pp13 and Pp16 formed biofilms and accumulated proline under stress conditions, while their phosphorus-solubilizing activity was only slightly affected by elevated salinity and pH. Notably, Pp13 maintained IAA-like metabolite production and ACC deaminase activity under stress conditions. Inoculation with either strains significantly improved the growth and stress tolerance of foxtail millet under saline-alkaline conditions. Bacterial inoculation increased proline accumulation, elevated ascorbate peroxidase activity, improved photosynthetic performance, and reduced oxidative damage and sodium accumulation, resulting in greater biomass accumulation.

**Discussion:**

These findings demonstrate that Pp13 and Pp16 possess multiple stress-adaptive and plant growth-promoting traits that contribute to improved crop performance under saline-alkaline conditions. The results highlight the potential of these strains as bioinoculants for sustainable crop production in saline-alkaline soils and identify *Kocuria* species as a relatively unexplored source of beneficial microorganisms for enhancing plant stress tolerance.

## 1 Introduction

Saline-alkaline stress has become a major environmental constraint limiting agricultural productivity worldwide. According to the Global Map of Salt-Affected Soils released by the Food and Agriculture Organization of the United Nations (FAO), more than 60% of soils are affected by salinity, while over 5% of soils are affected by saline-alkaline conditions (https://www.fao.org/global-soil-partnership/gsasmap/en/). These stresses alter soil physicochemical properties, reduce nutrient availability, and ultimately decrease crop productivity (Gangwar et al., 2020; Daba and Qureshi, 2021).

Saline-alkaline soils are characterized by excessive accumulation of soluble salts and/or carbonate, resulting in elevated soil electrical conductivity and pH. High salt concentrations reduce soil water potential and induce osmotic stress, thereby restricting water uptake by plants. In addition, high soil pH decreases nutrient availability, disrupt cellular pH homeostasis, and impairs enzyme activity and membrane integrity. Consequently, saline-alkaline stress generally exerts more severe effects on plant growth and development than salinity alone (Fang et al., 2021). To survive under such conditions, plants have evolved various adaptive mechanisms, including regulation of stomatal aperture, accumulation of osmoprotectants, and activation of antioxidant defense systems to mitigate oxidative damage (Fang et al., 2021). However, the effectiveness of these responses varies depending on plant species, developmental stage, and the intensity and duration of stress exposure (Balasubramaniam et al., 2023; Liu et al., 2024).

The reclamation and sustainable utilization of saline-alkaline soils have therefore become urgent challenges for global agriculture. Conventional approaches, such as chemical amendment and irrigation management, can alleviate soil salinity but are often costly and environmentally unsustainable. In contrast, breeding stress-tolerant cultivars is a long-term and labor-intensive process. Increasing evidence suggests that plant-associated microorganisms offer an environmentally friendly and sustainable alternative for improving plant performance under saline-alkaline conditions (Etesami and Maheshwari, 2018; Orhan, 2021). Among them, halotolerant plant growth-promoting bacteria (PGPB) have attracted considerable attention because they can survive under stressful environments through mechanisms such as extracellular polymeric substance production and can enhance plant growth by producing phytohormones, fixing nitrogen, and improving nutrient availability.

In our previous study, six PGPB strains isolated from the endosphere of Napier grass (*Pennisetum purpureum* Schum.) significantly promoted the growth of foxtail millet. These bacterial isolates were identified by comparing their 16S rRNA gene sequences with those in the NCBI Genebank database using BLAST. These strains exhibited multiple plant growth-promoting traits, including phosphorus and zinc solubilization, siderophore production, free nitrogen fixation, indole-3-acetic acid (IAA) production, and 1-aminocyclopropane-1-carboxylate (ACC) deaminase activity (Ko et al., 2024). In the present study, we first evaluated the salt tolerance of these bacterial isolates and selected *Kocuria palustris* Pp13 (Pp13) and *Bacillus aryabhattai* Pp16 (Pp16) for further investigation of their saline-alkaline tolerance and their potential to enhance plant stress tolerance.

Members of the genus *Bacillus* have been extensively reported to alleviate saline-alkaline stress and improve plant growth in diverse plant species (Zhou et al., 2017; Khoso et al., 2024; Xu et al., 2026; Zhou et al., 2026). In contrast, the plant growth-promoting potential and stress-mitigating functions of *Kocuria* species remained largely unexplored. Among the reported species, *Kocuria rhizophila* has demonstrated the ability to enhance plant growth and mitigate the detrimental effects of salinity and heavy metal stress (Hussain et al., 2019; Li et al., 2020; Almalkawi et al., 2025). However, limited information is available regarding the plant growth-promoting characteristics and stress tolerance mechanisms of other *Kocuria* species. Therefore, the present study aimed to characterize the plant growth-promoting traits of selected bacterial strains under different saline-alkaline conditions and to evaluate their effectiveness in enhancing saline-alkaline stress tolerance in foxtail millet Setaria italica (L.) P. Beauv.

## 2 Materials and methods

### 2.1 Bacteria tolerance to saline-alkaline stress

Bacteria were cultured in Luria-Bertani broth (LB) medium at 30°C overnight, subcultured into fresh LB medium, and adjusted to an optical density at 600 nm (OD_600_) of 1.0, corresponding to approximately 1×10^9^ and 5×10^8^ CFU mL^-1^ for Pp13 and Pp16, respectively. To evaluate tolerance to saline-alkaline stress, solid LB media were prepared with different combinations of NaCl concentration (1, 5, and 10%, w/v) and pH (7, 8, 9, and 10). Bacterial suspension (10 μL) was spotted onto agar plates.

### 2.2 Evaluation of bacteria physiological traits

Bacterial strains were cultured in LB medium under the saline-alkaline conditions described in Section 2.1.

Biofilm formation was determined using the microtiter plate method described by O'Toole et al. (1999) was used with minor modifications. Bacterial strains were cultured in LB medium at 30°C for 24h. The cells were harvested and resuspended in fresh LB medium, and adjusted to an OD_600_ = 0.3. Aliquots of bacterial suspensions (200 μL) were dispensed into the wells of a 96-well microtiter plate and incubated at 30°C for 72 h to allow biofilm formation. After incubation, the culture medium was carefully removed, and the attached biofilm was stained with 125 μL of 0.1% (w/v) crystal violet for 10 min. The well was then rinsed gently with ddH_2_O to remove extra stain. The bound crystal violet was solubilized with 30% (v/v) acetic acid for 15 min, and the resulting solution was transferred to a new microtiter plate. Biofilm formation was quantified by measuring the absorbance at 595 nm.

For proline determination, 6 mL of bacterial culture was centrifuged at 8,000 ×g for 5 min. The pellet was resuspended in 1 mL of 3% (w/v) sulfosalicylic acid and was added to resuspend the cells and stayed at room temperature for 24 h. After centrifugation at 13,000 ×g for 5 min, 250 μL of supernatant was mixed with 250 μL of glacial acetic acid and 250 μL of ninhydrin agent (0.3 g ninhydrin dissolved in 7.2 mL glacial acetic acid and 4.8 mL 6 M phosphoric acid). The reaction mix was heated in boiling water for 1 h and immediately cooled on ice. Subsequently, 1 mL of toluene was added and the mixture was vortexed and incubated in the dark for 20 min. Absorbance of the toluene phase was measured at 520 nm. Proline concentration was calculated using a standard curve (Garcia et al., 2017).

Phosphate-solubilizing ability was determined according to the method of Nautiyal (1999) with minor modifications. Bacterial suspensions (100 μL, OD_600_ = 1) were inoculated into NBRIP medium containing different combinations of NaCl concentration (1%, 5% and 10% NaCl) and pH (pH 7, pH 8, and pH 9) and incubated at 30°C for 4 d. Cultures were centrifuged, and the supernatant was filtered through 0.45 μm membrane. Solubilized phosphate was quantified using the vanadomolybdate method by measuring absorbance at 420 nm and calculating phosphate concentration from a standard curve.

IAA-like metabolite production was determined according to the method of Gordon and Weber (1951) with minor modifications. Bacterial suspensions (200 μL, OD_600_ = 1) were inoculated into minimal salt (MSL) medium (pH 7 or pH8) supplemented with 5 mM L-tryptophan and incubated at 30°C. Culture samples were collected at 24, 48 and 72 h after inoculation and centrifuged for 1 min. Subsequently, 60 μL of the supernatant was mixed with 120 μL of Salkowski reagent and incubated in the dark for 30 min. Absorbance was measured at 530 nm using a microplate reader. IAA-like metabolite concentrations were quantified using a standard curve generated with pure IAA.

ACC deaminase activity was measured following the method of Dworkin and Foster (1958) with minor modifications. Bacterial strains were cultured overnight at 30°C in LB medium containing 1%, 5% or 10% NaCl. Cells were harvested by centrifugation, washed three times with Dworkin and Foster (DF) medium containing the corresponding NaCl concentration, and resuspended in DF medium supplemented with the same NaCl concentration and 3 mM ACC. After incubation for 24h, bacterial cells were collected and resuspended in 300 μL of 0.1 M Tris-HCl buffer (pH 8.5), followed by the addition of 15 μL toluene. The toluene layer (100 μL) was mixed with 10 μL 0.5 M ACC and incubated at 30°C for 15 min. The reaction was terminated by adding 500 μL of 0.56 M HCl and centrifuging the mixture. Subsequently, 500 μL of the suspension was mixed with 400 μL of 0.56 M HCl and 150 μL of 0.2% 2,4-dinitrophenylhydrazine and incubated for 30 min. The reaction was stopped by adding 1 mL of 2 N NaOH, and the absorbance was measured at 540 nm. The enzyme activity was quantified based on α-ketobutyrate production using a standard curve generated with α-ketobutyrate. Protein concentration in the toluene layer was determined using the Bradford’s assay (Bradford, 1976), and ACC deaminase activity was expressed as μmol α-ketobutyrate (α-kbt) mg^-1^ protein h^-1^.

### 2.3 Plant growth conditions and saline-alkali stress treatment

Foxtail millet (*Setaria italica* (L.) P. Beauv.) cultivar Taitung 8 (TT8) was used in this study. Seeds were heat-treated at 45°C for 30 min and surface-sterilized with 2% (v/v) NaOCl. After germination of 1 week, seedlings were transplanted into nursery trays containing sterilized river sand and grown in a controlled-environment chamber under a 12-h light (28°C)/12-h dark (22°C) photoperiod with 60% relative humidity. After 2 weeks, seedlings were transplanted into 3-inch pots filled with substrates of three saline-alkaline levels (pH 7.7, EC 3.5 dS m^-1^; pH 7.8, EC 4.3 dS m^-1^; and pH 7.8, EC 6.6 dS m^-1^) and cultivated for additional 2–3 weeks.

Plants were fertilized twice a week with half-strength Hoagland’s nutrient solution at 7 mL per nursery pot and 15 mL per 3-inch pot. The nutrient solution contained 2.5 mM Ca(NO_3_)_2_. 7H_2_O, 2.5 mM KNO_3_, 1 mM MgSO_4_. 7H_2_O, 0.05 mM NaFeEDTA, 0.2 mM KH_2_PO_4_, 10 μM H_3_BO3, 0.2 μM Na_2_MoO_4_. 2H_2_O, 1 μM ZnSO_4_. 7H_2_O, 2 μM MnCl_2_. 4H_2_O, 0.5 μM CuSO_4_. 5H_2_O, 0.2 μM CoCl_2_. 6H_2_O and 0.5 mM 2-(N-morpholino)ethanesulfonic acid (MES). On non-fertilization days, an equivalent volume of distilled H_2_O was applied. Bacterial inoculants were applied once per week as described in Section 2.4, whereas mock-treated plants received an equal volume of sterile distilled H_2_O. Shoot and root samples were harvested for subsequent analyses.

Saline-alkaline soils (pH 8.0; electrical conductivity 9.6 dS m^-1^) was collected from an agricultural field in Beimen District, Tainan, Taiwan (N 23.24733˚, E 120.13067˚). To establish different stress intensities, the soil was mixed with sterilized river sand to generate mild stress conditions (pH 7.7, EC 3.5 dS m^-1^), moderate stress conditions (pH 7.8, EC 4.3 dS m^-1^), and severe stress conditions (pH 7.8, EC 6.6 dS m^-1^). To evaluate the intrinsic tolerance of TT8, plants were grown under these conditions for 2 weeks, and the wilting index was assessed according to the Standard Evaluation System for Rice developed by the International Rice Research Institute (IRRI, 2002). Plants were subsequently harvested for determination of biomass and relative water content. To assess the effects of bacterial inoculation on stress tolerance, plants were grown under the mild saline-alkaline stress condition and harvested after 3 weeks of treatment for analyses of osmotic adjustment and oxidative stress responses.

### 2.4 Bacteria inoculation

*Kocuria palustris* Pp13 and *Bacillus aryabhattai* Pp16 was cultured overnight in LB medium at 30°C. Bacterial cells were collected, resuspended in sterilized ddH_2_O, and adjusted to an OD_600_ of 1, corresponding to approximately 1×10^9^ and 5×10^8^ CFU mL^-1^ for Pp13 and Pp16, respectively. Bacterial suspensions (OD_600_ = 1) were applied at 7 mL per nursery pot and 15 mL per 3-inch pot once per week until harvest.

### 2.5 Chlorophyll content and chlorophyll fluorescence

Leaf chlorophyll content was determined according to Kato and Shimizu (1987). Leaf tissue was homogenized in 50 mM sodium phosphate buffer (pH 6.8), followed by extraction with 95% (v/v) ethanol in the dark for 30 min. After centrifugation, absorbance of the supernatant was measured at 649 and 665 nm, corresponding to the maximum absorption wavelengths of chlorophyll *b* and chlorophyll *a*, respectively, using a spectrophotometer. Total chlorophyll content was calculated as:


$$\text{Total chlorophyll }\left(\text{mg }{\text{L}}^{-1}\right)=(6.1\;\times {\text{A}}_{665})+(20.04\;\times {\text{A}}_{649}).$$

Chlorophyll fluorescence was measured using Junior-PAM fluorometer (Heinz Walz GmbH, Germany). The effective quantum yield of photosystem II [Y(II)] was calculated as (F’m-F’)/(F’m). Where F’m is maximum fluorescence under actinic light and F’ is steady-state fluorescence.

### 2.6 Evaluation of plant osmotic stress responses

Relative water content (RWC) was determined from leaf fresh weight (FW), turgid weight (TW) and dry weight (DW). Fresh leaves were weighed immediately to obtain FW, rehydrated in distilled water overnight to determine TW, and dried at 65°C for 48h to determine DW. RWC was calculated as (FW-DW)/(TW-DW) × 100% (Smart and Bingham, 1974).

Leaf proline content was measured according to Bates et al. (1973). Approximately 100 mg of leaf tissues was extracted with 3% (w/v) sulfosalicylic acid. After centrifugation, 250 μL of the supernatant was mixed with 250 μL of glacial acetic acid and 250 μL of ninhydrin reagent. The mixture was heated in boiling water for 60 min, cooled on ice, and extracted with toluene. The absorbance of the toluene phase was measured at 520 nm.

### 2.7 Evaluation of plant oxidative stress responses

Electrolyte leakage was determined by incubating leaf samples in distilled water for 2 h and measuring the initial conductivity (EC1). Samples were then autoclaved at 121°C for 20 min, the final conductivity (EC2) was measured. Electrolyte leakage was calculated as (EC1/EC2) × 100%.

Peroxidase (POD) activities was measured according to Macadam et al. (1992). Approximately 150 mg of leaf tissues was homogenized in 50 mM potassium phosphate buffer (pH 5.8). The reaction mixture contained 50 mM potassium phosphate buffer (pH 5.8), 21.6 mM guaiacol and 39 mM H_2_O_2_, and absorbance was monitored at 470 nm. One unit of POD activity was defined as the amount of enzyme producing 1 μmol tetraguaiacol min^-1^.

Ascorbate peroxidase (APX) and catalase (CAT) activities were measured using enzyme extracts prepared from 100 mg of leaf tissues homogenized in 50 mM sodium phosphate buffer (pH 6.8). APX activity was determined according to Nakano and Asada (1981) using a reaction mixture containing 1.5 mM ascorbate, 0.75 mM NaFeEDTA, and 6 mM H_2_O_2_ in 150 mM potassium phosphate buffer (pH 7.0). The decrease in absorbance at 290 nm was monitored. One unit of APX activity was defined as the consumption of 1 μmol of ascorbate min^-1^. CAT activity was determined according to Aebi (1984) using 1 mM H_2_O_2_ in 100 mM sodium phosphate buffer (pH 7.0). One unit of CAT activity is defined as the decomposition of 1 μmol of H_2_O_2_ min^-1^.

### 2.8 Analysis of salt ion and nutrient accumulation

Dried leaf samples (50–100 mg) were digested in a mixed acid containing 70% HNO_3_ and 70% HClO_4_ at a ratio of 4:1 (v/v) at 200°C for 2–3 h. Concentrations of Na, K, Ca, Mg, Fe, Mn, Zn, Cu, and Al were determined using inductively coupled plasma optical emission spectrometry (ICP-OES).

### 2.9 Statistical analysis

All experiments were conducted with at least three independent biological replicates. Data were analyzed using R. Differences among treatments were evaluated by Student’s *t -*test or one-way or two-way analysis of variance (ANOVA) followed by least significant difference (LSD) test. Differences were considered statistically significant at *p* < 0.05.

## 3 Results

### 3.1 Evaluation of saline-alkaline tolerance of PGPB strains

The halotolerance of bacterial strains isolated from Napier grass was first evaluated. Among the 20 tested strains, most were able to grow in LB medium containing 5% NaCl, whereas only Pp3, Pp13, Pp16, Pp18 and Pp20 survived in medium containing 10% NaCl (**Supplementary Figures 1A, B**). Based on their plant growth-promoting traits and the effectiveness on plant growth promotion (Ko et al., 2024), Pp3, Pp13 and Pp16 were selected for further saline-alkaline tolerance assessment.

**Figure 1:**
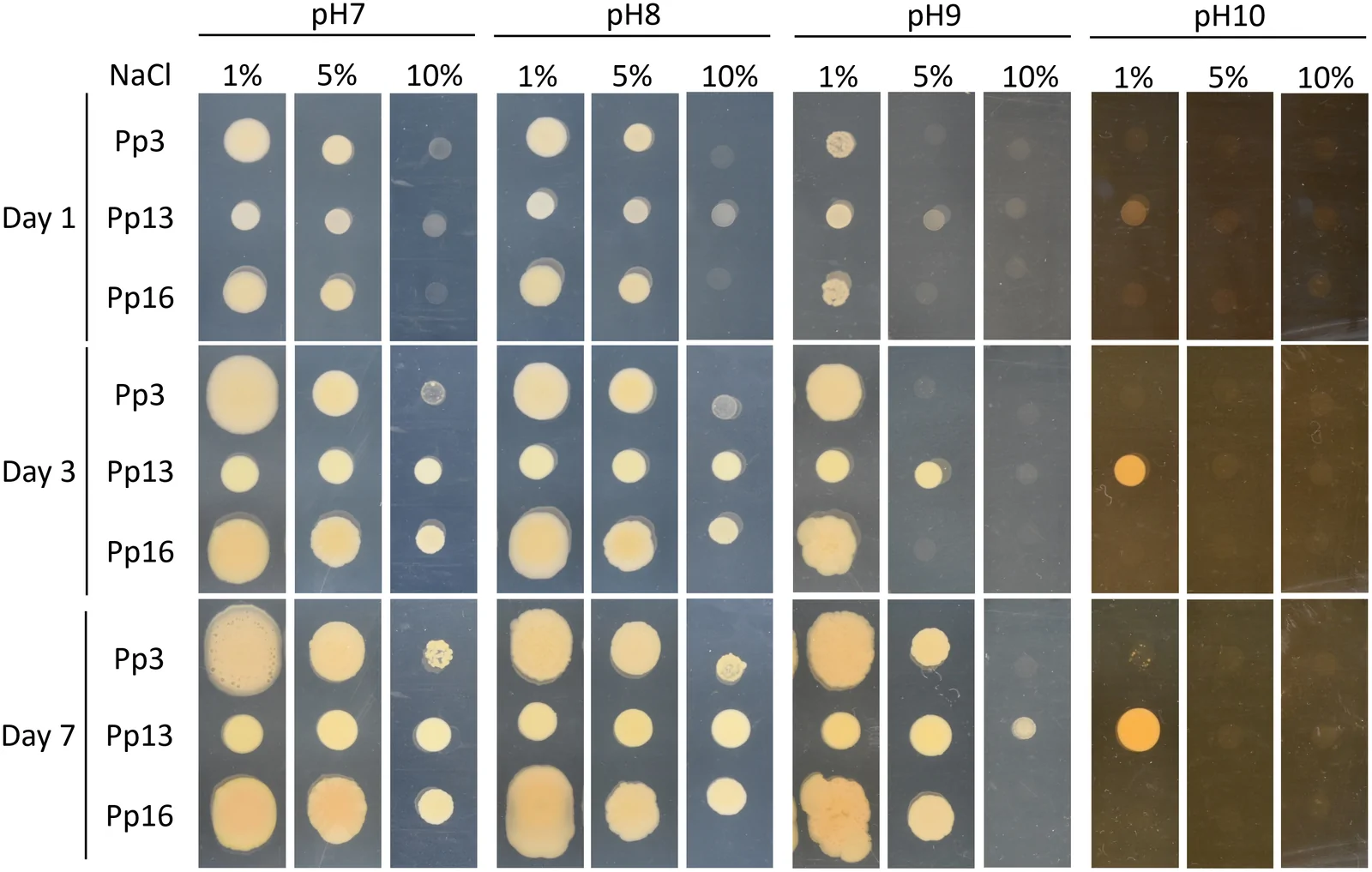
Growth of halotolerant strains under saline-alkaline conditions. Growth of Pp3, Pp13 and Pp16 in media containing 1%, 5% and 10% NaCl at pH 7-10. Representative images are shown.

All three strains were able to grow in medium containing up to 10% NaCl; however, increasing alkalinity markedly reduced bacterial viability. At pH 8, all three strains remained viable under all tested NaCl concentrations. At pH 9, only Pp13 survived in medium containing 10% NaCl, whereas at pH 10, Pp13 was the only strain capable of growth and only in medium containing 1% NaCl (**Figure 1**).

Because Pp13 and Pp16 exhibited relatively high tolerance to saline-alkaline conditions, these two strains were selected for further evaluation of salt tolerance and plant growth-promoting traits under stress conditions. Both strains were cultured in LB medium containing 1%, 5%, 10%, 12%, 15%, or 18% NaCl. Colonies of Pp13 and Pp16 were observed only in media containing up to 10% NaCl at 1 day after inoculation. At 6 day after inoculation, colonies of both strains were also observed in medium containing 12% NaCl, whereas bacterial growth was strongly inhibited at higher NaCl concentrations (**Supplementary Figure 1C**).

**Figure 2:**
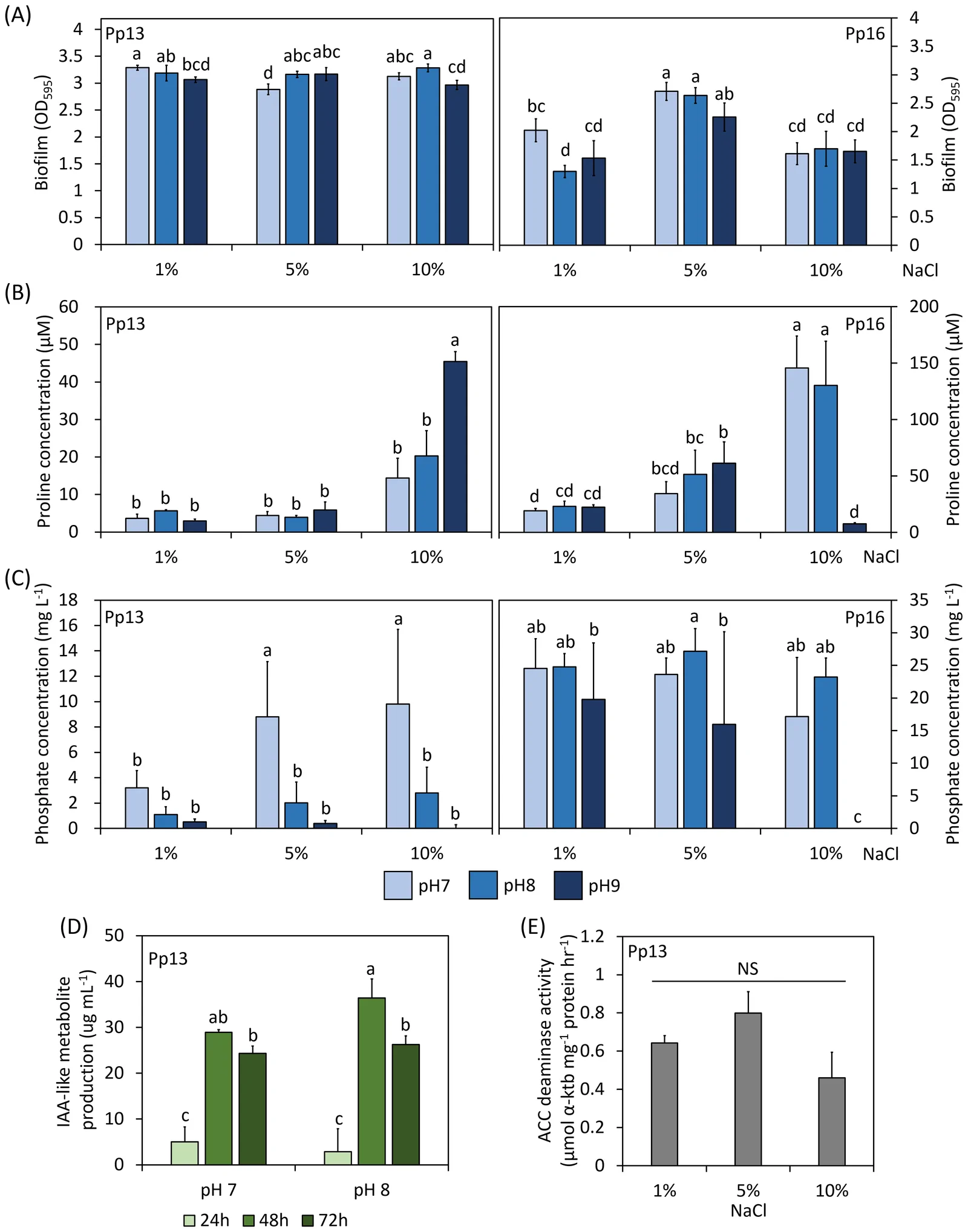
Plant growth-promoting traits of Pp13 and Pp16 under saline- alkaline conditions. **(A)** Biofilm formation. **(B)** Proline production. **(C)** Phosphorus-solubilizing activity. **(D)** IAA-like metabolite production by Pp13 at pH 7 and pH 8. **(E)** ACC deaminase activity of Pp13 under 1%, 5% and 10% NaCl conditions. Data are presented mean ± SD (n=3). Statistical differences were determined by ANOVA followed by LSD test. Different letters indicate significant differences among treatments (*p* < 0.05), whereas NS indicates no significant difference.

Biofilm formation by both Pp13 and Pp16 was quantified under different saline-alkaline conditions. In both strains, biofilm production was slightly decreased with increasing pH. However, Pp13 maintained relatively stable biofilm formation across different salinity levels, whereas biofilm production by Pp16 declined markedly as salinity increased (**Figure 2A**).

Proline production was also evaluated under saline-alkaline stress. At 1% and 5% NaCl, proline production by Pp13 and Pp16 remained relatively stable across the tested pH levels. Under 10% NaCl conditions, proline production by Pp13 increased significantly as pH increased. A similar trend was observed in Pp16, although proline production decreased significantly at pH 9, possibly because of reduced cell growth under severe stress conditions (**Figure 2B**).

Phosphorus-solubilizing activity was further assessed under saline-alkaline conditions. In Pp16, phosphorus-solubilizing activity was maintained under 10% NaCl conditions compared with that under 1% and 5% NaCl treatment; however, the amount of soluble phosphorus decreased significantly with increasing pH. In contrast, phosphorus solubilization by Pp13 was enhanced by increasing salinity but was inhibited by higher pH levels (**Figure 2C**).

IAA-like metabolite production was significantly reduced by saline-alkaline stress in both strains. In Pp16, IAA-like metabolite levels were below the detection limit under all tested stress conditions. Therefore, temporal change in IAA-like metabolite production was evaluated only for Pp13 cultured in medium containing 1% NaCl at pH7 or pH8. Under both conditions, IAA-like metabolite accumulation increased gradually over time and was not significantly affected pH, although a slight decrease was observed at 72 h after inoculation (**Figure 2D**).

ACC deaminase activity was strongly affected by alkaline stress and was therefore evaluated only under 1%, 5% and 10% NaCl conditions. Despite a substantial reduction in Pp13 growth under high-salt conditions, ACC deaminase activity at 10% NaCl remained comparable to that at 1% NaCl, with no significant difference observed (**Figure 2E**).

### 3.2 Effects of Pp13 and Pp16 on the growth of foxtail millet under saline-alkaline conditions

**Figure 3:**
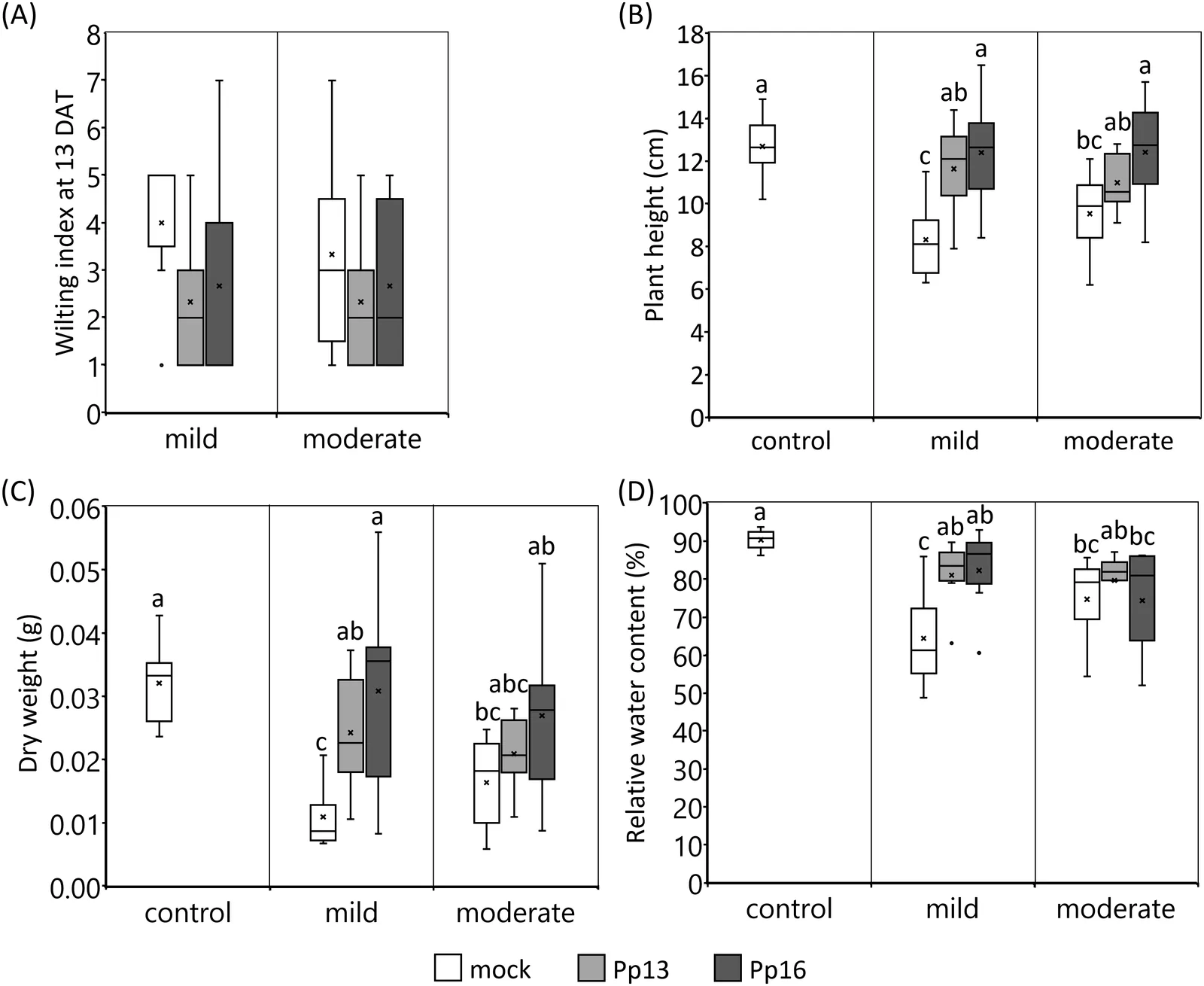
Effects of saline-alkaline stress on foxtail millet seedling growth. **(A)** Wilting index at 13 days after treatment (DAT). **(B)** Plant height. **(C)** Shoot dry weight. **(D)** Relative water content. Box plots show the median (center line), first and third quartiles (box boundaries). The x-axis indicates the saline-alkaline stress treatments and the color key is shown below the panels. Statistical differences were determined by ANOVA followed by LSD test. Different letters indicate significant differences among treatments (*p* < 0.05) (n = 6).

To evaluate the potential of Pp13 and Pp16 on saline-alkaline tolerance in foxtail millet, mild, moderate, and severe stress conditions were established using different ratios of river sand and saline-alkaline soil. Two weeks after transplanting, all plants grown under severe stress conditions exhibited severe wilting and were therefore excluded from further analysis. Although wilting symptoms under mild and moderate stress conditions did not differ significantly, plants inoculated with Pp13 or Pp16 showed relatively lower wilting severity than mock-treated plants (**Figure 3A**, **Supplementary Figure 2**).

Plant height and dry weight were significantly reduced under both mild and moderate stress conditions compared with plants grown under control conditions. However, inoculation with Pp13 or Pp16 significantly improved plant growth under stress conditions (**Figures 3B, C**). A similar trend was observed for leaf relative water content. Under mild stress conditions, bacterial inoculation significantly increased relative water content, whereas under moderate stress conditions, the positive effects were less pronounced (**Figure 3D**). Because bacterial inoculation showed more significant effects under mild stress conditions, subsequent experiments were conducted under this condition.

**Figure 4:**
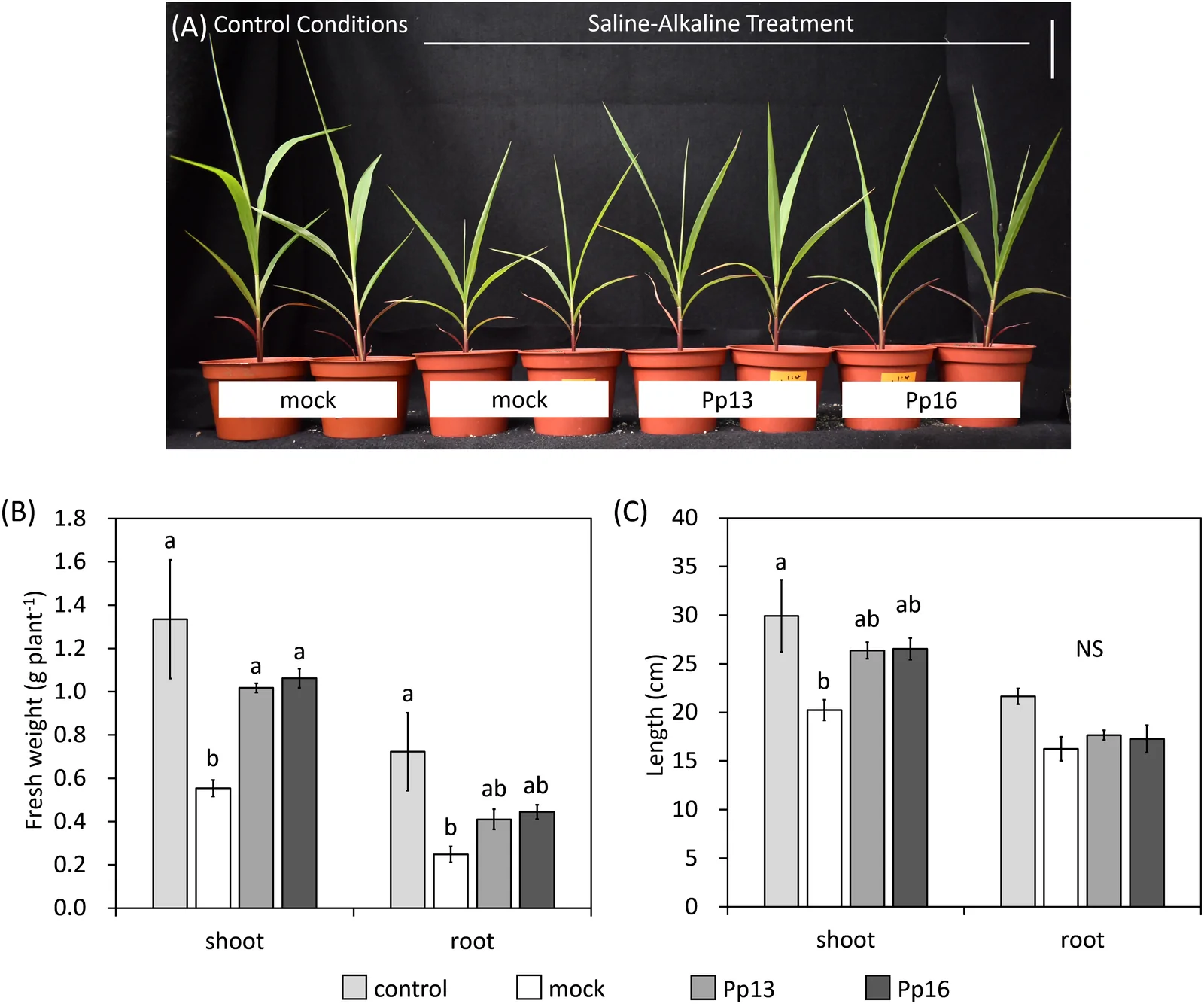
Growth performance of foxtail millet inoculated with bacterial isolates under mild saline-alkaline conditions. **(A)** Representative images of mock-treated and bacterial-inoculated plants grown under saline-alkaline treatments. **(B)** Fresh weight. **(C)** Shoot length and maximum root length. The control treatment represents mock-treated plants grown under non-stress conditions. Data are presented mean ± SE (n = 10-12). Statistical differences were determined by ANOVA followed by LSD test. Different letters indicate significant differences among treatments (*p* < 0.05), whereas NS indicates no significant difference.

Before transplantation into saline-alkaline soil, bacterial inoculation slightly increased plant height by approximately 16% compared with mock treatment. At 7 days after transplanting, saline-alkaline treatment significantly inhibited around 15% of plant growth compared with plants grown under normal conditions. At this stage, growth promotion by Pp13 and Pp16 inoculation led to plant height comparable to that of control plants, and the positive effects became more pronounced at 21 days after transplanting (**Figure 4A**; **Supplementary Figures 3**, **4**). Consistent with the differences in plant height, shoot fresh weight was highest in plants grown under control conditions. Saline-alkaline stress significantly reduced shoot fresh weight in mock-treated plants, whereas inoculation with Pp13 or Pp16 increased shoot fresh weight by approximated 1.8-to-1.9-fold, resulting in biomass comparable to that of control plants (**Figures 4B, C**).

Root growth was also evaluated. Although maximum root length was not significantly affected by saline-alkaline treatments, root volume was markedly reduced under stress conditions (**Supplementary Figure 3**). Similarly, root fresh weight was lowest in mock-treated plants, whereas inoculation with Pp13 or Pp16 significantly increased root fresh weight by approximately 1.7-to-1.8-fold compared with the mock treatment (**Figures 4B, C**), indicating that both bacteria strains effectively alleviated the inhibitory effects of saline-alkaline stress on plant growth.

### 3.3 Effects of Pp13 and Pp16 on stress responses in foxtail millet

**Figure 5:**
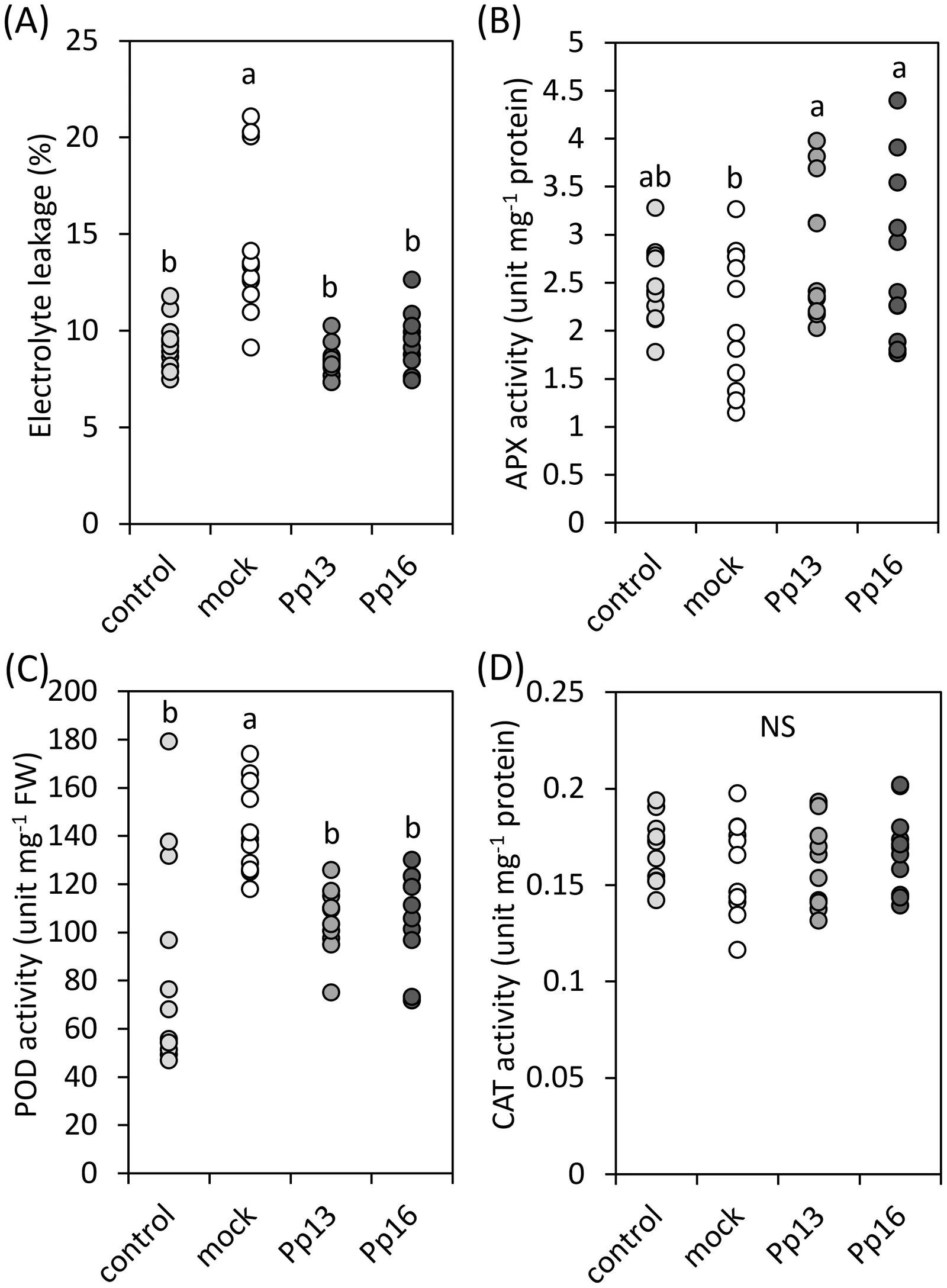
Oxidative damage and antioxidant enzyme activities in mock-treated and bacterial-inoculated foxtail millet under mild saline-alkaline stress. **(A)** Electrolyte leakage. **(B)** Ascorbate peroxidase (APX) activity. **(C)** Peroxidase (POD) activity. **(D)** Catalase (CAT) activity. The control treatment represents mock-treated plants grown under non-stress conditions. Statistical differences were determined by ANOVA followed by LSD test. Different letters indicate significant differences among treatments (*p* < 0.05) (n=11), whereas NS indicates no significant difference.

Saline-alkaline stress induced both oxidative stresses and osmotic stresses in plants. Oxidative stress disrupts cell membrane integrity, as reflected by increased electrolyte leakage. In this study, mock-treated plants exposed to saline-alkaline stress exhibited approximately 45% higher electrolyte leakage than those grown under control conditions. In contrast, electrolyte leakage in Pp13- and Pp16-inoculated plants under stress was comparable to that of mock-treated plants grown under control conditions (**Figure 5A**), suggesting that bacterial inoculation alleviated oxidative damage to cellular membrane.

Among the antioxidant enzymes examined, ascorbate peroxidase (APX) activity was significantly increased in Pp13- and Pp16-inoculated plants, whereas no significant change was observed in mock-treated plants grown under stress compared with those grown under control conditions (**Figure 5B**). In contrast, peroxidase (POD) activity was significantly elevated in mock-treated plants grown under stress but remained at control levels in inoculated plants (**Figure 5C**), indicating that Pp13 and Pp16 inoculation alleviated oxidative stress primarily through APX activation, thereby reducing the requirement for POD induction. Catalase (CAT) activity was not significantly affected by either saline-alkaline stress or by bacterial inoculation (**Figure 5D**).

**Figure 6:**
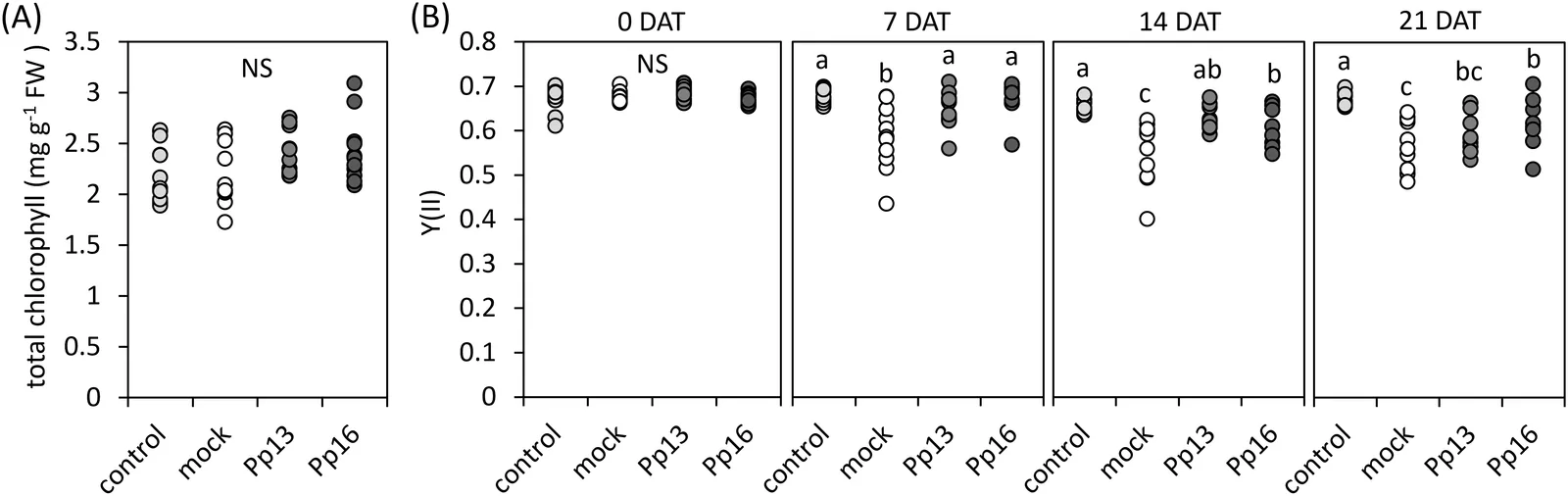
Photosynthetic performance of mock-treated and bacterial-inoculated foxtail millet under mild saline-alkaline stress. **(A)** Total chlorophyll content. **(B)** Effective quantum yield of photosystem II [Y(II)] at different time points after stress treatment. DAT, days after treatment. The control treatment represents mock-treated plants grown under non-stress conditions. Statistical differences were determined by ANOVA followed by LSD test. Different letters indicate significant differences among treatments (*p* < 0.05) (n = 10-12), whereas NS indicates no significant difference.

Although chlorophyll content was unaffected by saline-alkaline stress, the effective quantum yield of photosystem II [Y(II)] decreased significantly with prolonged stress exposure. Inoculation with Pp13 and Pp16 mitigate this decline, maintaining higher Y(II) values from 7 days after treatment (DAT) onward. Although prolonged stress exposure gradually reduced the beneficial effects, Y(II) value in inoculated plants remained higher than those in mock-treated plants (**Figure 6**).

**Figure 7:**
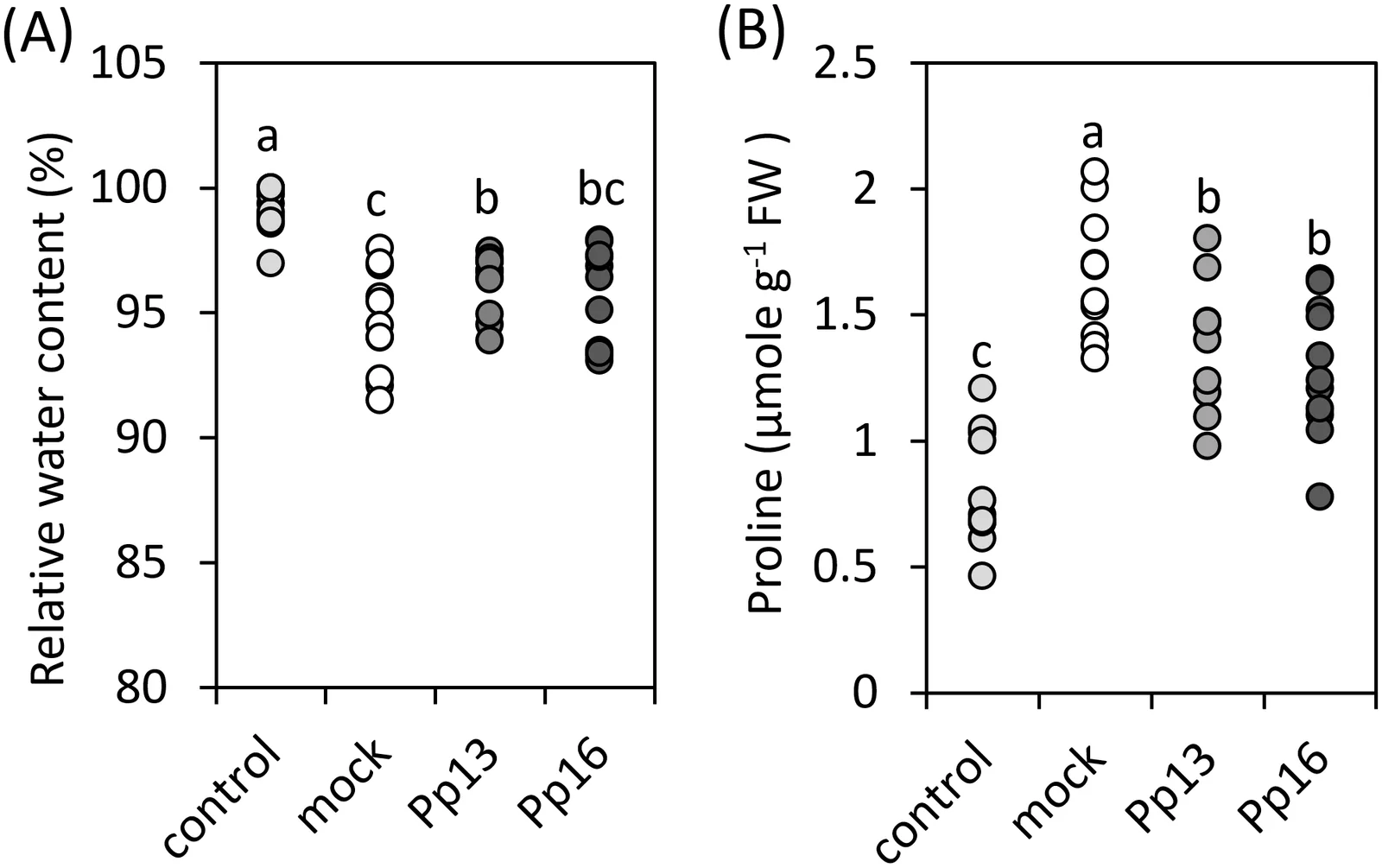
Osmotic stress responses of mock-treated and bacterial-inoculated foxtail millet under mild saline-alkaline stress. **(A)** Leaf relative water content. **(B)** Leaf proline concentration. The control treatment represents mock-treated plants grown under non-stress conditions. Statistical differences were determined by ANOVA followed by LSD test. Different letters indicate significant differences among treatments (*p* < 0.05) (n = 11).

Saline-alkaline treatment reduced leaf relative water content, whereas inoculation with Pp13 or Pp16 slightly increased water retention in plants (**Figure 7A**). Proline accumulation was also examined because proline serves as a major osmolyte under stress conditions. In mock-treated plants, proline concentration was significantly higher under stress than that under control conditions. In contrast, bacterial inoculation reduced proline accumulation (**Figure 7B**), likely due to alleviation of stress severity.

### 3.4 Effects of Pp13 and Pp16 on nutrient accumulation

**Figure 8:**
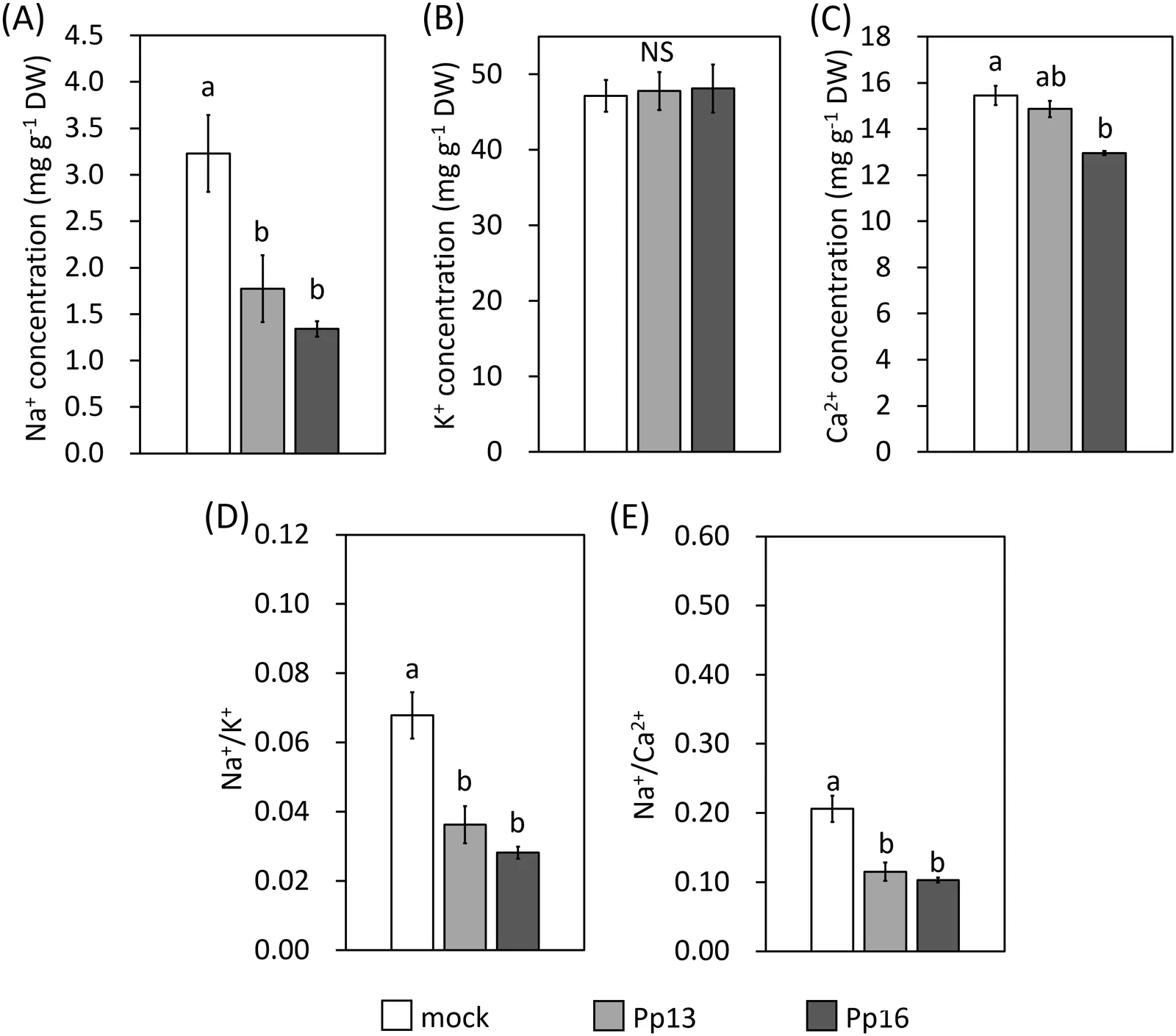
Leaf ion concentrations in mock-treated and bacterial-inoculated foxtail millet under mild saline-alkaline conditions. **(A)** Na^+^ concentration. **(B)** K^+^ concentration. **(C)** Ca^2+^ concentration. **(D)** Na^+^/K^+^ concentration. **(E)** Na^+^/Ca^2+^ concentration. Data are presented as mean ± SE (n = 6). Statistical differences were determined by ANOVA followed by LSD test. Different letters indicate significant differences among treatments (*p* < 0.05), whereas NS indicates no significant difference.

Excessive salt accumulation is a major factor causing cellular damages under saline-alkaline stress. Therefore, the effects of Pp13 and Pp16 on ion accumulation were investigated. Compared with the mock treatment, inoculation with either Pp13 or Pp16 reduced leaf Na^+^ concentration by approximately 50-60%, whereas K^+^ concentration was not significantly affected (**Figures 8A, B**). Consequently, Na^+^/K^+^ ratio was significantly lower in inoculated plants than in mock-treated plants (**Figure 8D**).

Unexpectedly, leaf Ca^2+^ concentration was also reduced following bacterial inoculation, decreasing by approximately 5% and 15% in Pp13- and Pp16-inoculated plants, respectively (**Figure 8C**). However, because the reduction in Na^+^ concentration was substantially greater than that in Ca^2+^, the Na^+^/Ca^2+^ ratio was remained lower in inoculated plants (**Figure 8E**).

The concentrations of other mineral elements, including Mg, Fe, Zn, Mn, Cu and Al, were also analyzed. Inoculation of Pp13 and Pp16 had little effect on the accumulation of these ions, although Zn and Cu concentrations were approximately 30% lower than those in mock-treated plants (**Supplementary Figure 5**). These results suggest that alleviation of saline-alkaline stress by Pp13 and Pp16 inoculation was primarily associated with reduced Na^+^ accumulation and improved ionic balance rather than enhanced mineral nutrient accumulation.

## 4 Discussion

Halotolerant PGPB are frequently isolated from saline environments or halophytic plants and often possess multiple plant growth-promoting traits, including nitrogen fixation, phosphorus solubilization, phytohormone production, ACC deaminase activity, which contribute to plant growth and stress adaptation (Etesami and Beattie, 2018). Rather than focusing on PGPB from extreme saline habitats, the present study evaluated bacterial strains isolated from the endosphere of Napier grass, a moderately salt-tolerant forage crop (Tsai et al., 2025). Among the tested strains, Pp13 and Pp16 exhibited the strongest tolerance to combined salinity and alkalinity, surviving in media containing up to 12% NaCl and elevated pH, although alkaline conditions reduced their salt tolerance (**Figure 1**; **Supplementary Figure 1**). Similar observation have been reported for halotolerant bacteria isolated from non-saline soils, suggesting that bacterial halotolerance is not exclusively associated with saline environments (Ait Bessai et al., 2023).

Compared with *Bacillus*, the plant growth-promoting potential of *Kocuria* species remains relatively under explored. Although some reported *Kocuria* strains have been isolated from saline habitats or arid environments, information regarding their roles in enhancing plant stress tolerance is still limited (Kim et al., 2004; Goswami et al., 2014; Li et al., 2025). Previous studies demonstrated that *K. rhizophila* Y1 tolerated up to 10% NaCl and enhanced maize salinity tolerance (Li et al., 2020). Consistent with these findings, Pp13 displayed strong tolerance to both salinity and alkalinity while retaining important plant growth-promoting traits under stress conditions (**Figures 1**, **2**). Like many previously reported *Kocuria* strains, Pp13 was capable of producing IAA-like metabolites (Hansda et al., 2017; Li et al., 2020; Afridi et al., 2021; Faddetta et al., 2023; Almalkawi et al., 2025). In addition to regulating plant growth, IAA has been implicated in bacterial adaptation to environmental stress (Duca and Glick, 2020). In the present study, Pp13 maintained the production of IAA-like metabolites under alkaline conditions and preserved phosphorus solubilizing and ACC deaminase activities under saline stress (**Figure 2**). Because IAA-like metabolites were quantified using a colorimetric assay, further confirmation by liquid chromatography-mass spectrometry is required to verify the identity and amount of bioactive IAA (Guardado-Fierros et al., 2024). Nevertheless, the maintenance of these characteristics under stress conditions may contribute to improved nutrient acquisition and stress mitigation in inoculated plants. Consistent with this hypothesis, Pp13- and Pp16-inoculated plants exhibited the reduced Na^+^ accumulation, greater biomass and attenuated stress responses compared with mock-treated plants (**Figures 4**, **8**). Collectively, our results expand current knowledge of *Kocuria* species and highlight their potential as beneficial microorganisms for saline-alkaline agriculture.

Both Pp13 and Pp16 maintained biofilm formation and accumulated proline under stress conditions (**Figure 2**), suggesting that these responses may contribute to bacterial adaptation to osmotic stress. Biofilms are primarily composed of extracellular polymeric substances (EPS), which protect bacterial cells from dehydration and toxic ions while improving soil water retention and ion sequestration (Kasotia et al., 2016; Costa et al., 2018; Grinev et al., 2020; Bhagat et al., 2021; Daud et al., 2023; Kim et al., 2024). In addition, EPS-producing bacteria have been reported to alleviate abiotic stress in plants by enhancing osmotic adjustment, antioxidant capacity, and water-use efficiency (Naseem and Bano, 2014; Sun et al., 2020).

Although EPS production was not directly quantified in this study, the ability of Pp13 and Pp16 to form biofilm under saline-alkaline conditions suggested that EPS may contribute to their beneficial effects on plant stress alleviation. This interpretation is consistent with the marked reduction in leaf Na^+^ accumulation observed in inoculated plants (**Figure 8A**), which may reflect improved ion exclusion or immobilization in the rhizosphere. However,the relationship between biofilm formation and enhanced plant stress tolerance remains to be verified. Future studies using purified EPS, EPS- or biofilm-deficient mutants will help to clarify the role of bacterial biofilms in promoting saline-alkaline stress tolerance and further support the potential use of Pp13 and Pp16 as bioinoculants for saline-alkaline soils.

Oxidative stress is a major consequence of saline-alkaline stress, resulting from excessive accumulation of ROS. In the present study, inoculation with Pp13 and Pp16 significantly enhanced APX activity accompanied by reduced electrolyte leakage (**Figure 5**), indicating alleviation of oxidative damage. APX is a key component of the ascorbate-glutathione cycle and has a higher affinity for H_2_O_2_ than CAT and POD, making it particularly important for ROS detoxification under stress conditions (Gill and Tuteja, 2010). Enhanced APX activity has widely reported as a mechanism underlying PGPB-mediated stress tolerance in plants exposed to drought and salinity (Giannelli et al., 2023). In contrast, POD activity was lower in inoculated plants than in mock-treated controls (**Figure 5C**). Antioxidant responses are known to vary depending on plant genotype, stress severity, stress duration and plant-microbe interactions (Neto et al., 2006; Torun, 2019; Sarker and Oba, 2020; Azeem et al., 2023; Tiwari et al., 2024; Shirvani et al., 2026). Therefore, the reduced POD activity observed in inoculated plants may reflect a lower oxidative burden resulting from more efficient ROS scavenging through APX-mediated pathways. Together, these findings suggest that modulation of antioxidant defense systems is an important mechanism by which Pp13 and Pp16 enhance plant tolerance to saline-alkaline stress.

Although Pp13 and Pp16 possessed multiple stress-tolerant traits and PGP properties and effectively alleviated saline-alkaline stress in foxtail millet, the mechanism underlying their interaction with the host plant were unclear. Because bacterial re-isolation from the rhizosphere or endosphere was not performed, we could not determine whether the observed stress alleviation resulted primarily from direct plant-bacterial interactions or from indirect effects, such as transient rhizosphere activity or the production of bacterial metabolites. Future studies incorporating bacterial tracking and colonization assays are needed to verify the establishment and persistence of Pp13 and Pp16 *in planta* and to elucidate the mechanisms by which they enhance plant tolerance to saline-alkaline stress.

## 5 Conclusion

This study identified two halotolerant PGPB strains, *K. palustris* Pp13 and *B. aryabhattai* Pp16, that exhibited strong tolerance to salinity and alkalinity while maintaining key plant growth-promoting traits. Both strains accumulated biofilms and proline under stress conditions, which likely contributed to their adaptation to saline-alkaline environments. Pp13 and Pp16 retained phosphorus-solubilizing activity under salinity stress, while Pp13 additionally maintained IAA-like metabolite production and ACC deaminase activity. Inoculation with either strain improved photosynthetic performance, enhanced osmolyte accumulation, reduced Na^+^ accumulation and alleviated oxidative damage in foxtail millet grown under saline-alkaline conditions. These beneficial effects were associated with modulation of antioxidant enzyme activities, particularly increased APX activity. Notably, this study provides evidence supporting the potential of *Kocuria* species as PGPB for improving crop performance under saline-alkaline stress. The findings highlight Pp13 and Pp16 as promising bioinoculants for sustainable crop production in salt-affected soils.

## Data Availability

The original contributions presented in the study are included in the article/**Supplementary Material**. Further inquiries can be directed to the corresponding author/s.
