# Supplementary material for: Halotolerant Kocuria and Bacillus species enhanced saline-alkaline stress tolerance in foxtail millet
Source: Front Plant Sci. 2026 Aug 19;17:1910313. doi: 10.3389/fpls.2026.1910313 (PMC13533941; doi:10.3389/fpls.2026.1910313)
Supplement: Supplementary file 1 [file Image1.pdf]

Supplementary Material

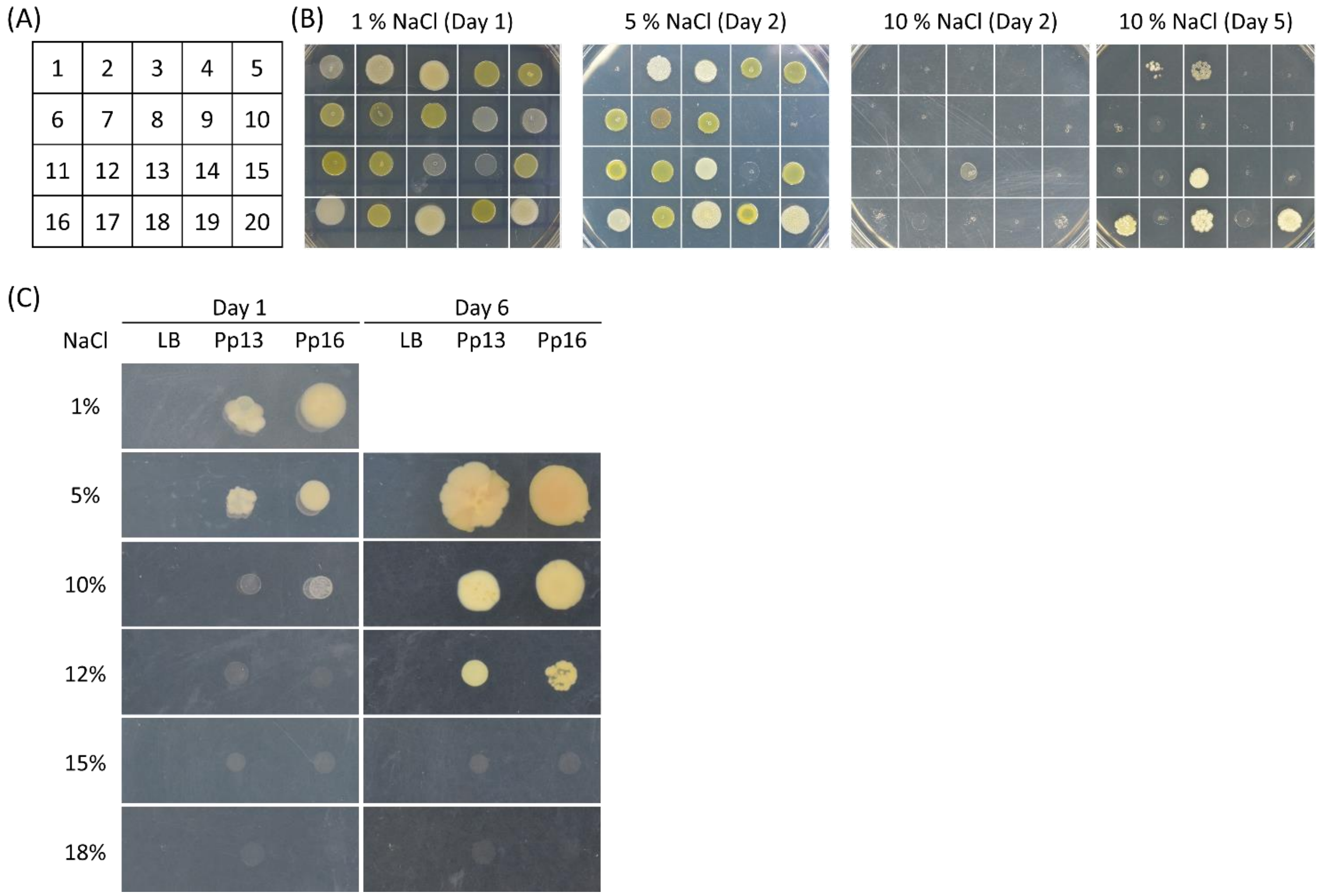

Supplementary Figure 1. Salt tolerance of bacterial isolates obtained from the endosphere of Napier grass.

(A) Arrangement of bacterial isolates on culture medium. (B) Growth of 20 bacterial isolates on media supplemented with 1% or 5% or 10% NaCl. (C) Growth of Pp13 and Pp16 under different NaCl concentrations. Experiments were independently repeated three times, and representative images were shown.

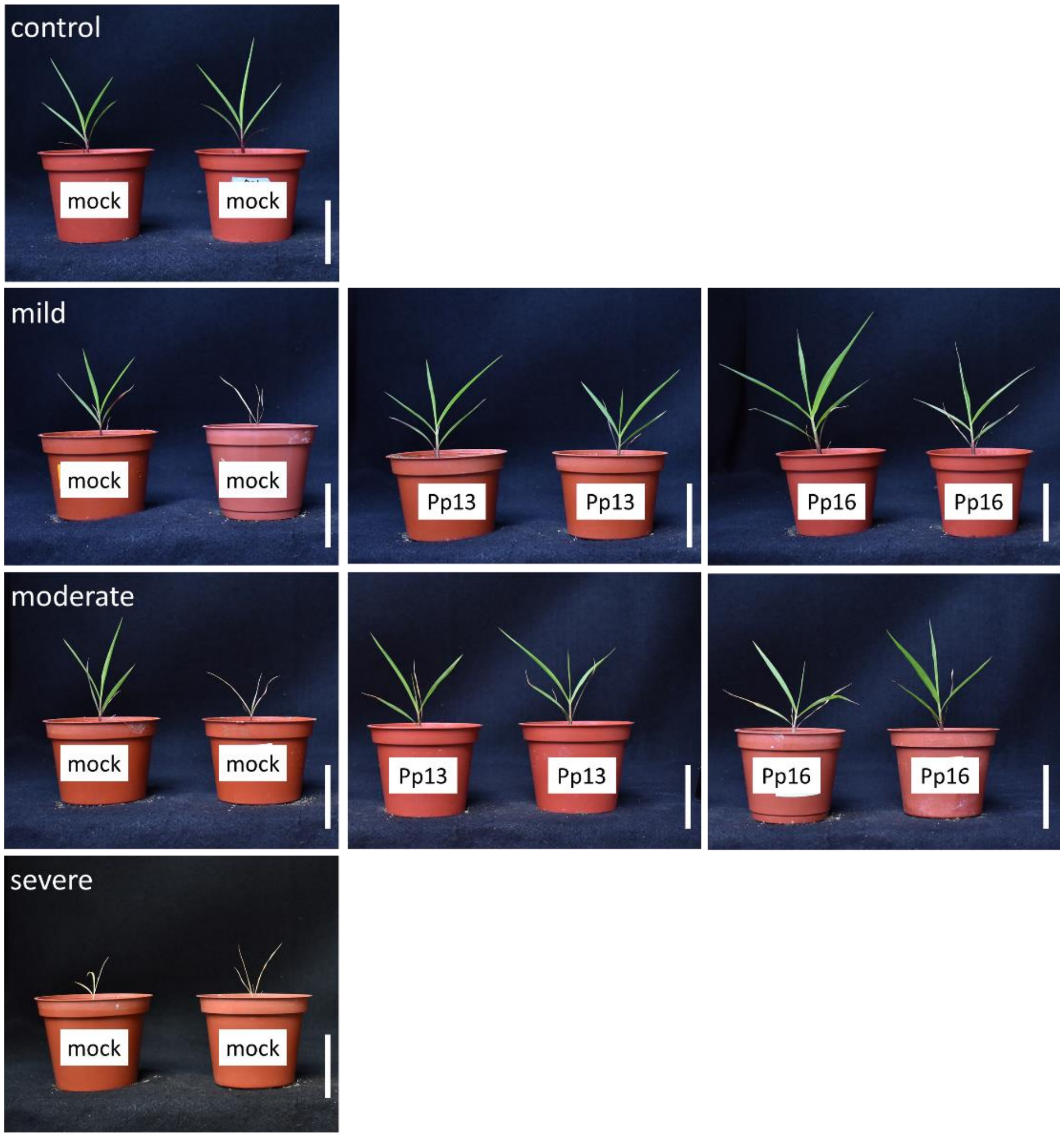

Supplementary Figure 2. Growth performance of foxtail millet under different levels of saline-alkaline conditions.

Representative images of plants grown under control, mild, moderate, or severe saline-alkaline stress conditions. Scale bar = 5 cm.

(A)

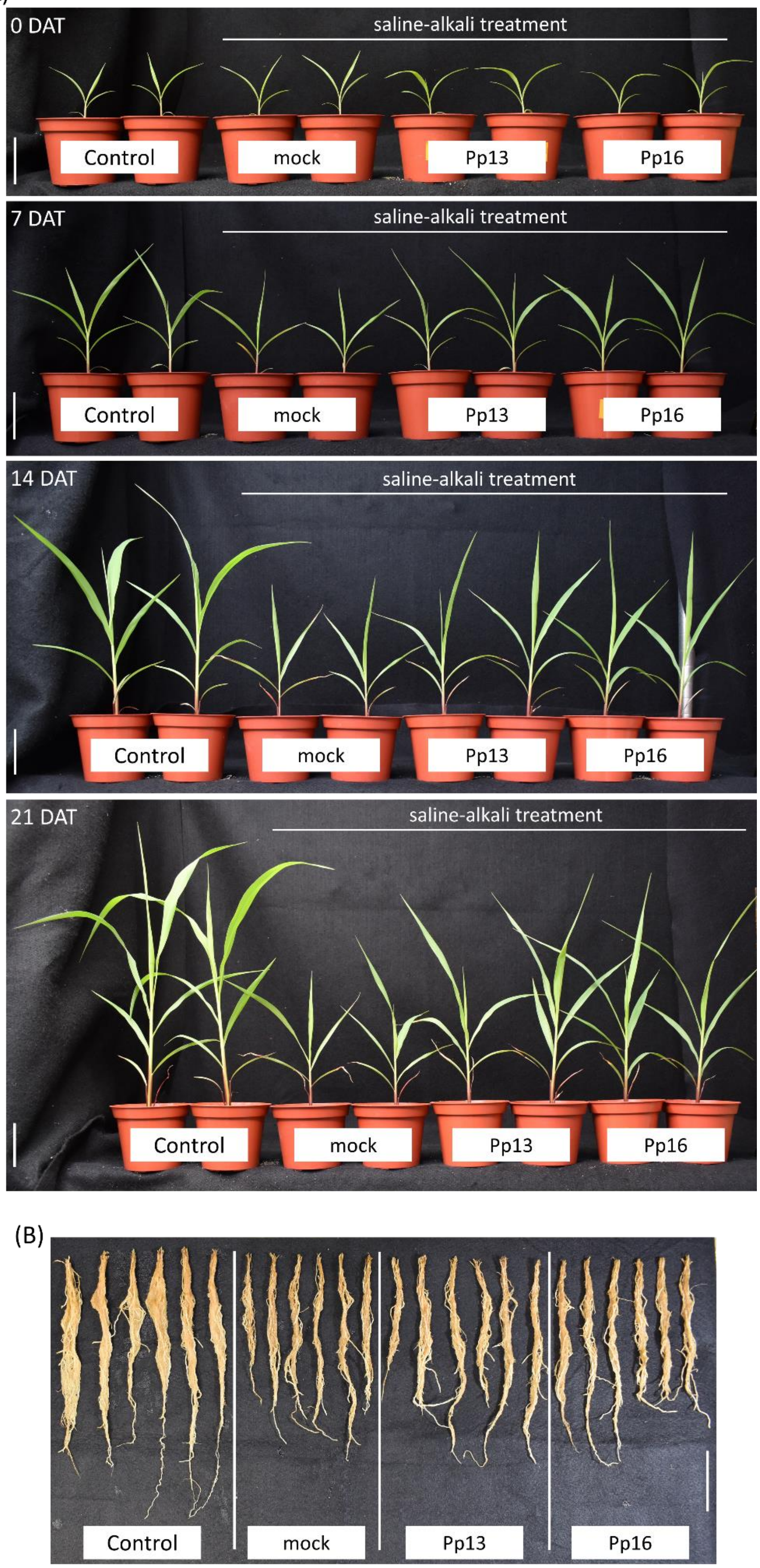
0 DAT
saline-alkali treatment
Control
mock
Pp13
Pp16
7 DAT
saline-alkali treatment
Control
mock
Pp13
Pp16
14 DAT
saline-alkali treatment
Control
mock
Pp13
Pp16
21 DAT
saline-alkali treatment
Control
mock
Pp13
Pp16
(B)
Control
mock
Pp13
Pp16

Supplementary Figure 3. Shoot and root growth of foxtail millet under control and mild stress conditions.

(A) Shoot growth, (B) Root growth. DAT, days after treatment. Scale bar = 5 cm.

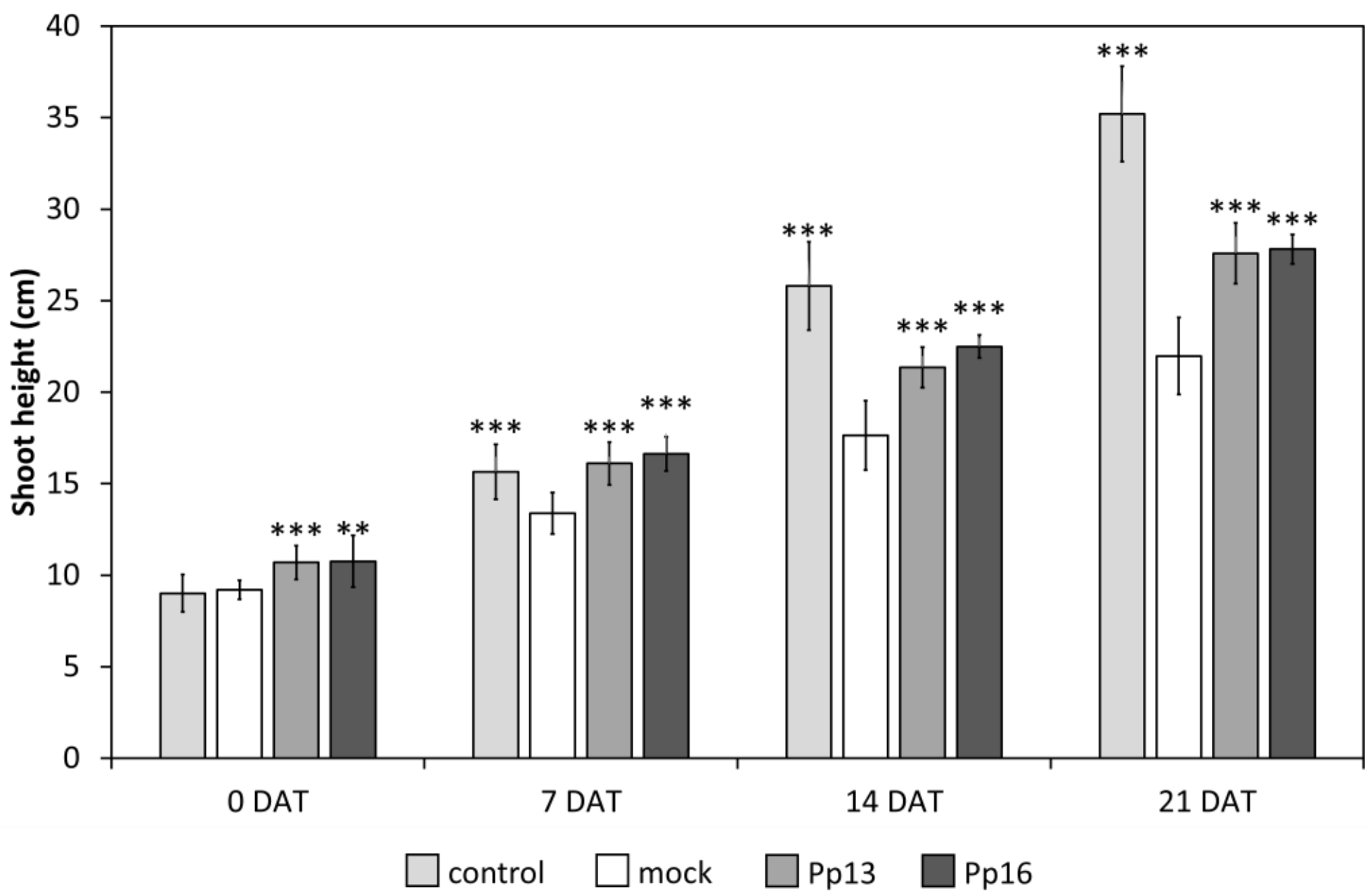

Supplementary Figure 4. Changes in shoot height of mock-treated and bacterial-inoculated foxtail millet during mild saline-alkaline stress treatment.

Shoot height was measured at different time points after stress treatment. DAT, days after treatment. Data are presented as mean ± SD (n = 10-12). Differences between mock treatments grown under stress and control conditions, as well as mock-treated and bacterial-inoculated plants under saline-alkaline stress, were assessed using Student's *t*-test. ***, p<0.001.

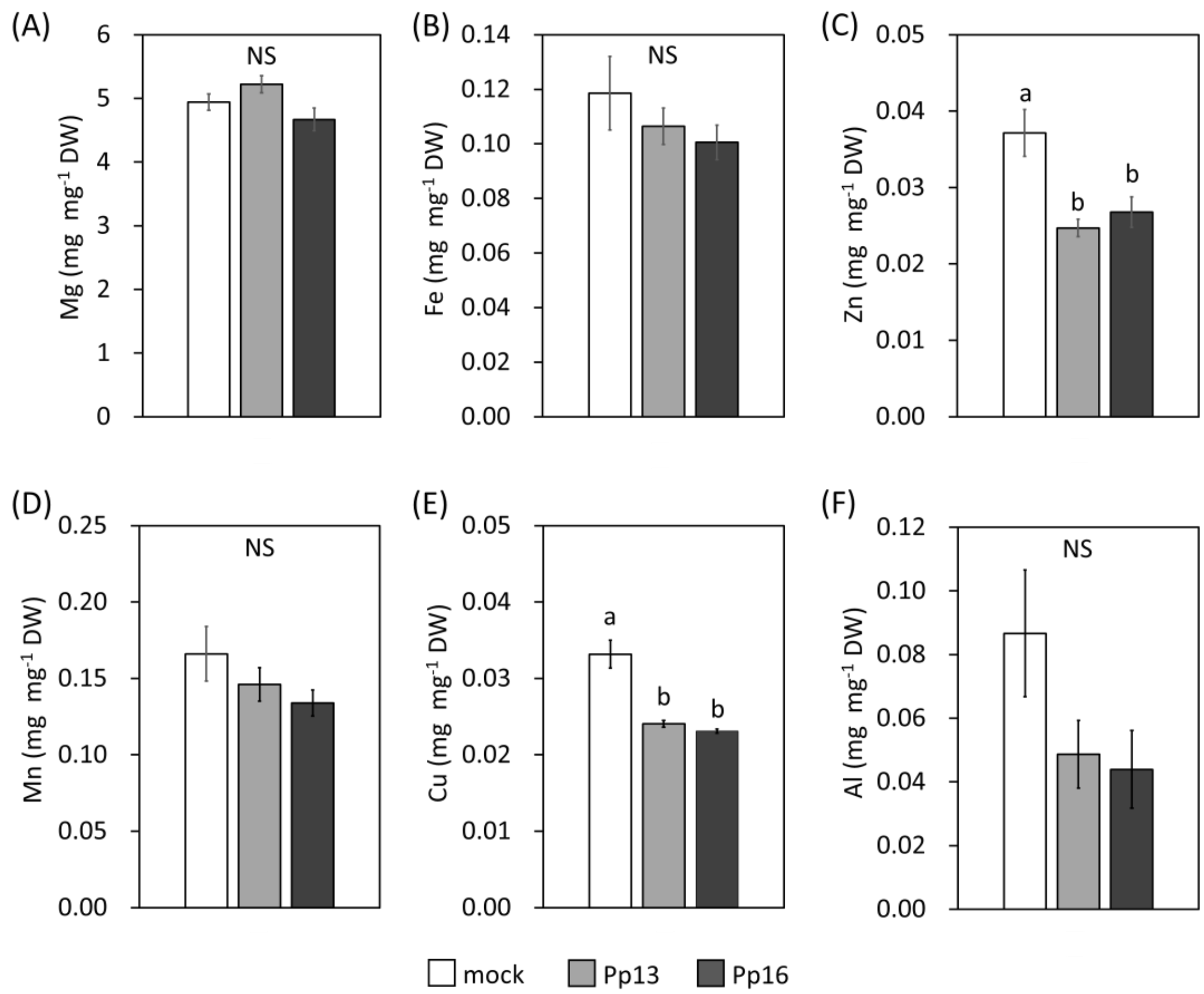

Supplementary Figure 5. Leaf mineral ion concentrations in mock-treated and bacterial-inoculated foxtail millet under mild saline-alkaline stress.

(A) Mg concentration. (B) Fe concentration. (C) Zn concentration. (D) Mn concentration. (D) Cu concentration. (E) Al concentration. Data are mean ± SE (n=6). Statistical differences were determined by ANOVA followed by LSD test. Different letters indicate significant differences among treatments ($p<0.05$), whereas NS indicates no significant difference.
